# Eculizumab use in patients with pneumococcal-associated hemolytic uremic syndrome and kidney outcomes

**DOI:** 10.1007/s00467-023-06037-2

**Published:** 2023-06-12

**Authors:** Patrik Konopásek, Jakub Zieg

**Affiliations:** 1 Department of Pediatric Nephrology, 2nd Faculty of Medicine, University Hospital Motol, Charles University, Prague, Czech Republic; 2 Pediatrická Klinika 2. LF UK and FN v Motole, V Úvalu 84, Prague 5, 15006 Czech Republic

**Keywords:** *Streptococcus pneumoniae*, Hemolytic uremic syndrome, Eculizumab, Chronic kidney disease, Dialysis

## Abstract

**Background:**

*Streptococcus pneumoniae*-associated hemolytic uremic syndrome (P-HUS) is a rare and severe disease. Only a few reports have been published about eculizumab use in P-HUS.

**Methods:**

We analyzed demographic, clinical, and laboratory data of patients with P-HUS from our center.

**Results:**

The cohort consisted of 4 females and 3 males. All patients had pneumonia. Four were given eculizumab (days 1–3). The eculizumab group required a shorter duration of dialysis and mechanical ventilation (medians 20 vs. 28.5 and 30 vs 38.5 days, respectively) compared with the non-eculizumab group, but this was still much longer than normally reported; the thrombocytopenia resolution was similar in both groups (medians 10 vs. 8 days). Chronic kidney disease (CKD) was correlated with the duration of dialysis and mechanical ventilation duration at 1 year (*r* = 0.797, *P* = 0.032 and *r* = 0.765, *P* = 0.045) and last follow-up (*r* = 0.807, *P* = 0.028 and *r* = 0.814, *P* = 0.026, respectively); our scoring system showed even stronger correlations (*r* = 0.872, *P* = 0.011 and *r* = 0.901, *P* = 0.0057, respectively). The eculizumab group showed slightly better 1-year and last follow-up CKD stage (2.75 vs. 3, *P* = 0.879 and 2.5 vs. 3.67, *P* = 0.517).

**Conclusions:**

Despite the fact that the eculizumab group showed better outcomes, eculizumab does not seem to improve the course of P-HUS compared with previous reports. Kidney outcomes are strongly correlated with the duration of dialysis and mechanical ventilation duration.

**Graphical Abstract:**

**Figure:**
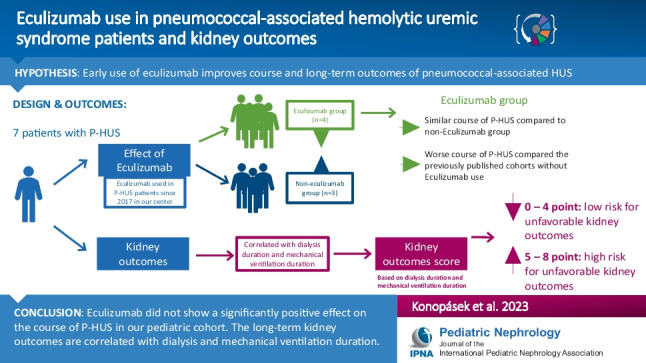
A higher resolution version of the Graphical abstract is available as Supplementary information

**Supplementary Information:**

The online version contains supplementary material available at 10.1007/s00467-023-06037-2.

## Introduction

Hemolytic uremic syndrome (HUS) is characterized by thrombocytopenia, microangiopathic hemolytic anemia, and acute kidney injury (AKI). HUS associated with Shiga-toxin producing *Escherichia coli* (STEC-HUS) is the most common form, accounting for about 85–90% of cases in children [1, 2].

*Streptococcus pneumoniae*-associated HUS (P-HUS, also abbreviated as SP-HUS or Sp-HUS by some authors) accounts for about 5% of all HUS cases and is associated with a more severe course, while long-term kidney outcomes seem to be similar to STEC-HUS. The risk factors most strongly associated with chronic kidney disease (CKD) are related to the severity of the initial kidney injury and the need for acute dialysis for longer than 20 days [2–5].

It has been thought that neuraminidase activity resulting in the desialylation of red blood cell membrane glycoproteins leading to exposure of the Thomsen-Friedenreich cryptantigen (T-antigen) is the principal mechanism of developing P-HUS [3]. Recent papers suggest that T-antigen exposure is not sufficient to explain the pathophysiology of the disease [1]. Other factors like abnormal complement activation in individuals with genetic variations in complement pathway regulation variations may play a critical role [1, 6, 7]. Symptomatic treatment is the pillar in P-HUS, although other approaches like intravenous immune globulin (IVIG), plasma exchanges (PE), and complement blockade by anti-complement C5 monoclonal antibody eculizumab have been used [1].

This study aimed to describe the course and kidney outcomes in our patients with P-HUS.

## Methods

We analyzed all patients with P-HUS diagnosed and treated in our center from 2009 to 2022. HUS was defined as thrombocytopenia, AKI, and microangiopathic hemolytic anemia. The presence of confirmed pneumococcal infection (pneumonia, sepsis, or meningitis and positive culture and/or polymerase chain reaction from blood, sputum, cerebrospinal fluid, and/or pleural effusion or positive pneumococcal urine antigen test) was necessary for the P-HUS diagnosis. CKD was defined as abnormalities of kidney structure or function, present for 3 months. Eculizumab treatment was indicated in all patients with P-HUS and a sign of alternative complement pathway activation (decreased C3) since 2017. The decision for the initiation and duration of the treatment was individually made by the consultant nephrologist as there was no clear evidence in the literature to guide the treatment. All relevant information was searched in the patient’s medical records. Electronic hospital records provided all necessary data (Table 1). Genetic analyses for complement abnormalities were not performed because the logistics of the biological material and further evaluation in the laboratory which is situated out of the Czech Republic has not been covered by the insurance in children with P-HUS. The glomerular filtration rate was estimated by the Schwartz formula [8]. *T*-tests were used to compare the distributions of the continuous variables and Pearson’s correlation coefficient (*r*) was measured to explore the association between continuous variables. A* P*-value ≤ 0.05 was considered statistically significant.

**Table 1:** Patient information

Patient	1	3	5	6	–	2	4	7
Parameters at the disease onset								
Sex	F	F	F	F	–	M	M	M
Year of P-HUS onset	2018	2017	2018	2020	–	2016	2011	2013
Age at P-HUS onset (years)	0.70	5.59	0.96	3.62	–	2.84	1.76	0.80
Pneumococcal vaccination	10-valent	No	No	No	–	13-valent	No	Yes, the type NA
Type of infection	PN	PN	PN	PN	–	PN	PN	PN
Pneumococcal diagnosis	PCR + in pleural effusion	PUAT +	PCR +	PUAT +	–	Type 3, pleural effusion cultivation	PUAT +	PUAT +
Coombs test	Negative	Positive	Positive	Positive	–	Positive	Positive	Positive
T-antigen	NA	Positive	NA	NA	–	Positive	NA	Positive
Time from PN Dg to P-HUS Dg (days)	2	0	2	5	–	1	2	2
C3 (g/l)/day after P-HUS Dg	0.62/1	0.24/1	0.43/1	0.51/0	–	0.63/0	0.68/2	0.91/0
C4 (g/l)/day after P-HUS Dg	0.12/1	0.03/1	0.14/1	0.12/0	–	0.13/0	0.16/2	0.11/0
Maximum LDH (µkat/l) at P-HUS onset	156	74.4	118.98	99.6	–	93.94	76.6	113.41
Clinical parameters								
Time from P-HUS Dg to dialysis initiation (days)	1	1	0	1	–	0	0	0
Type of dialysis	CVVHD	CVVHD	CVVHD	CVVHD	–	CVVHD	PD	CVVHD—PD
Duration of dialysis (days)	18	17	78	22	–	43	5	36
Duration of intubation (days)	26	20	60	31	–	35	0	42
Time to PLT normalization	8	8	11	8	–	10	5	36
Time to LDH normalization	44	34	23	15	–	13	20	76
PE	No	Yes	No	No	–	Yes	No	Yes
Time from P-HUS Dg to Eculizumab treatment (days)	1	3	1	1	–	Not used	Not used	Not used
Eculizumab doses	3	7	2	3	–	Not used	Not used	Not used
Need for transfusion	Yes	Yes	Yes	Yes	–	Yes	Yes	Yes
RDP	Yes	No	No	Yes	–	No	No	No
Pleural effusion	Bilateral	Left side	Bilateral	Left side	–	Left side	Small – left side	Left side
Effusion drainage	Yes	No	Yes	Yes	–	Yes	No	No
Hemithoracoscopy	Yes	No	Yes	Yes	–	Yes	No	No
Lobectomy	Yes	No	No	Yes	–	Yes	No	No
Catecholamines use	Yes	Yes	Yes	Yes	–	Yes	No	Yes
Parameters during follow-up								
Follow-up (years)	4.60	1.00	4.75	1.84	–	6.75	6.72	9.75
C3 (g/l) last follow-up	0.89	1.00	1.17	1.21	–	1.07	0.79	1.11
C4 (g/l) last follow-up	0.15	0.2	0.49	0.36	–	0.21	0.18	0.2
GFR (ml/min/1.73 m^2^) on 1-year follow-up	115	132	PD	29	–	PD	117	31
CKD on 1-year follow-up	1	1	5	4	–	5	1	3
GFR on last follow-up	99	132	79 (Tx)	35.9	–	43 (Tx)	121	74 (Tx)
Kidney outcome score	3	2	7	4	–	5	1	5
Risk classification	Low risk	Low risk	High risk	Low risk	–	High risk	Low risk	High risk

*CKD* chronic kidney disease, *CVVHD* continuous venovenous hemodialysis, *Dg* diagnosis, *GFR* glomerular filtration rate, *LDH* lactate dehydrogenase, *NA* not available, *P-HUS* pneumococcal-associated hemolytic uremic syndrome, *PCR* polymerase chain reaction, *PD* peritoneal dialysis, *PE* plasma exchange, *PLT* platelets, *PN* pneumonia, *PUAT* pneumococcal urine antigen test, *RDP* platelet concentrate, *Tx* kidney transplant

C3 normal ranges: 0.83–2.25 g/l, C4 normal ranges: 0.14–0.35 g/l, GFR normal ranges: 90–130 ml/min/1.73 m^2^

## Results

### Baseline characteristics

**Fig. 1:**
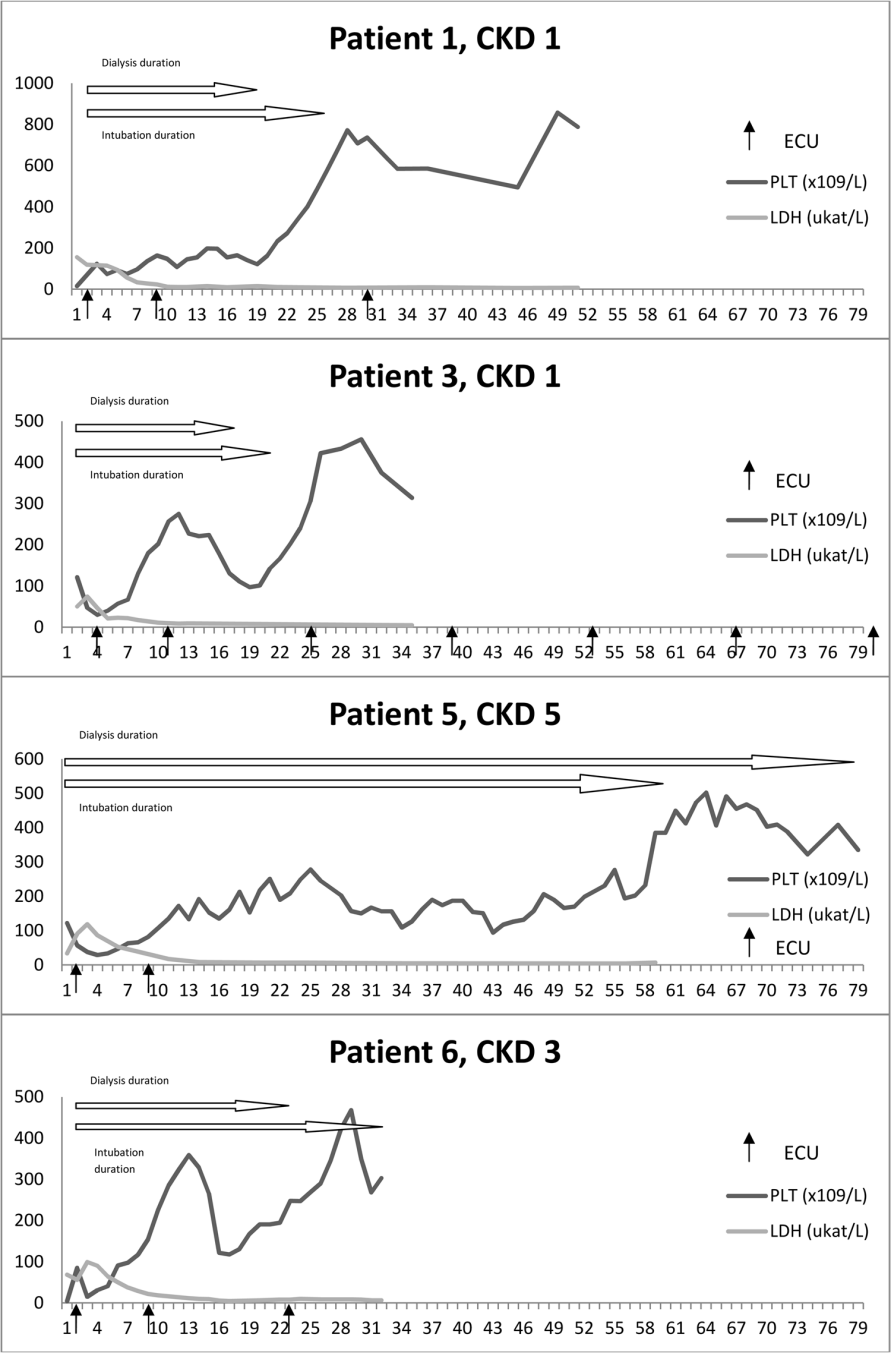
Timelines of the patients treated with eculizumab including the last follow-up CKD. *CKD*, chronic kidney disease; *ECU*, eculizumab application; *LDH*, lactate dehydrogenase; *PLT*, platelets

**Fig. 2:**
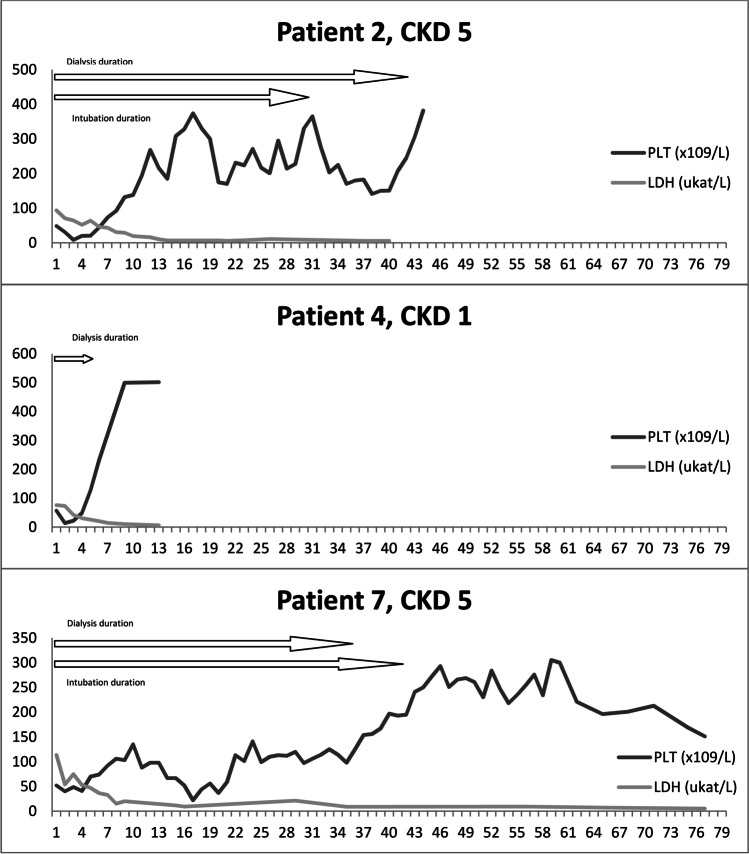
Timelines of the patients not treated with eculizumab including the last follow-up CKD. *CKD*, chronic kidney disease; *LDH*, lactate dehydrogenase; *PLT*, platelets

The cohort consisted of 3 males and 4 females (8.64% of all HUS cases). The mean age of P-HUS onset was 2.32 ± 1.8 years, and the mean follow-up was 5.04 ± 3.05 years. All patients were diagnosed with pneumonia; the course was severe in the majority of them, with a mean dialysis and mechanical ventilation (MV) duration of 31.3 ± 24.15 and 29.29 ± 18.91 days respectively, and a need for lobar pneumonectomy in 3 cases. Only 3 patients were vaccinated with pneumococcal conjugated vaccine prior to pneumonia. Continuous venovenous hemodialysis (CVVHD) was the most commonly used kidney replacement therapy (KRT). Two children were treated with PE and 3 with eculizumab, and another with eculizumab and concomitant PE. Platelets (PLT) reached normal values in a median of 8 days (5–36 days) and lactate dehydrogenase (LDH) in a median of 23 days (13–76 days). All children required KRT (5–78 days) and all but one MV (0–60 days). Three patients reached CKD stage 5 and underwent kidney transplantation (Table 1, Figs. 1 and 2).

### Kidney outcomes

Only KRT and MV duration were associated with poor kidney outcomes. There was a positive correlation between the duration of both KRT and MV and CKD grade at 1-year follow-up (*r* = 0.797, *P* = 0.032 and *r* = 0.765, *P* = 0.045, respectively) and last follow-up (*r* = 0.807, *P* = 0.028 and *r* = 0.814, *P* = 0.026, respectively). Based on that, we created a scoring system to predict long-term kidney outcomes. Patients were given points for KRT duration (0 days—0 points, 1–20 days—1 point, 21–40 days—2 points, 41–60 days—3 points, > 60 days—4 points) and MV duration (the same scoring system). Patients with 0–4 points were graded as low risk and 5–8 points as high risk for unfavorable kidney outcomes. The score was strongly correlated with CKD at 1 year and the last follow-up (*r* = 0.872, *P* = 0.011 and *r* = 0.901, *P* = 0.0057, respectively) (Table 1, Figs. 1 and 2).

### Eculizumab vs. non-eculizumab group

Patients who received eculizumab had a slightly lower C3 complement level and higher maximal LDH measured at the moment of P-HUS diagnosis, but the results did not reach statistical significance (0.45 vs. 0.74 g/l, *P* = 0.061 for C3 and 112.25 vs. 94.65 µkat/l, *P* = 0.427), C3 was not obtained on the same day after P-HUS diagnosis (days 0–2) in all cases, and also LDH reached maximum levels on different days. The eculizumab group required KRT for 17–78 days (median 20) and MV for 20–60 days (median 28.5); in the non-eculizumab group, the duration of KRT was 5–43 days (median 36) and MV was 0–42 days (median 38.5) days. It took a similar time to normalize PLT in both groups (medians 10 vs. 8 days) and to reach normal LDH (median 20 vs. 28.5 days, respectively). Eculizumab was given 1–3 days after P-HUS diagnosis (Table 1). The eculizumab group showed a slightly better 1-year and last follow-up CKD stage without statistical significance (2.75 vs. 3, *P* = 0.879 and 2.5 vs. 3.67, *P* = 0.517, respectively) (Figs. 1 and 2).

## Discussion

We report a small case series of patients with a high occurrence of severe courses of P-HUS. Four of them received early eculizumab which did not seem to improve the course of the disease, even when C3 complement levels were low. The duration of KRT and MV was correlated with CKD grade, which led us to create a scoring system that showed a strong correlation with kidney outcomes.

Few case reports have been published about the use of eculizumab in P-HUS. All authors noticed improvements related to complement blockade. See et al. used eculizumab in a 2-year-old girl on the third day after P-HUS diagnosis. The PLT level started to rise after 3 days (day 6 after P-HUS), and she required KRT for 15 days in total [9]. Jeantet et al. used eculizumab in a 55-year-old man after unsuccessful PE. Eculizumab was given on the 7th day after P-HUS manifestation, and the PLT level normalized on day 13 (6 days after eculizumab application); the patient required dialysis for 21 days [10]. Gilbert et al. gave eculizumab to a 21-month-old female on the 7th day of P-HUS, reporting an improvement in PLT level 12 h after eculizumab application; however, prior to the application, PLT count was already 138 × 10^9^/l. KRT was needed for 30 days [11]. Holle et al. published the only case series with the use of eculizumab in 3 patients. They required KRT for 21–45 days and the authors reported a rapid improvement in PLT and LDH levels in two of them, but the information about the day of eculizumab application and duration of thrombocytopenia was not clear [12]. We believe that previous authors overestimated the possible positive effect of eculizumab. Two case series reported a median of 6 days of thrombocytopenia and dialysis duration of 5–25 (median 16) days and 0–20 (median 13) days in P-HUS without eculizumab therapy [13, 14]. In a large series of patients from the UK, the median duration of KRT was 10 days [3]. Therefore, almost all the cases treated with eculizumab, including our patients, required a longer KRT than normally reported and thrombocytopenia did not improve more quickly than compared to children not treated by complement blockade. The median duration of KRT and MV was indeed shorter, and the kidney outcomes were slightly better in the eculizumab group compared to the non-eculizumab group in our cohort, but we believe that the difference was only random rather that due to eculizumab use because the patients had still worse outcomes than in the previous reports without eculizumab. All patients had low complement levels and eculizumab was used early; therefore, the expected outcomes should have been much better for KRT duration and PLT resolution. Based on our results, the administration of a complement blockade did not significantly improve the course of P-HUS in our cohort, although the number of patients was small. Further investigation is needed to explore whether some subgroups of P-HUS patients could benefit from the treatment, such as those with genetic variations in complement pathway regulation [6, 7].

It was previously reported that children with P-HUS requiring dialysis for more than 20 days had a higher risk of developing CKD [4]. In patients with STEC-HUS, the duration of anuria and prolonged dialysis are the most important factors of poor kidney outcomes [15]. In our study, the duration of KRT and MV were associated with poor kidney outcomes; based on that, we created a scoring system that strongly correlated with kidney outcomes. Proper symptomatic treatment including antibiotic therapy from the beginning seems to be of major importance for patients with P-HUS.

The small number of patients is the main limitation of our series. The course of the disease in the eculizumab group could be biased by the increased complement activation status based on slightly lower C3 levels and more active disease (higher LDH). On the other hand, the possible higher activity of the disease must be taken with caution because the results did not reach statistical significance. In addition, C3 and maximum LDH levels were not obtained at the same time during the course of the disease. Despite that, our work brings a new view on eculizumab use in P-HUS as this is the largest cohort with this treatment reported so far. It also supports a previously reported association between dialysis duration and kidney outcomes.

In conclusion, despite the fact that the eculizumab group had slightly better outcomes, eculizumab did not show a significantly positive effect on the course of P-HUS in our pediatric cohort. The long-term kidney outcomes are correlated with dialysis and MV duration.

### Supplementary Information

Below is the link to the electronic supplementary material.

Graphical abstract (PPTX 47 KB)

## Data Availability

All data generated or analyzed during this study are included in this published article.
