# Supplementary material for: Eculizumab use in patients with pneumococcal-associated hemolytic uremic syndrome and kidney outcomes
Source: Pediatr Nephrol. 2023 Jun 12;38(12):4209–15. doi: 10.1007/s00467-023-06037-2 (PMC10584715; doi:10.1007/s00467-023-06037-2)
Supplement: Supplementary file 1 — Graphical abstract (PPTX 47 KB) [file 467_2023_6037_MOESM1_ESM.pptx]

## Slide 1
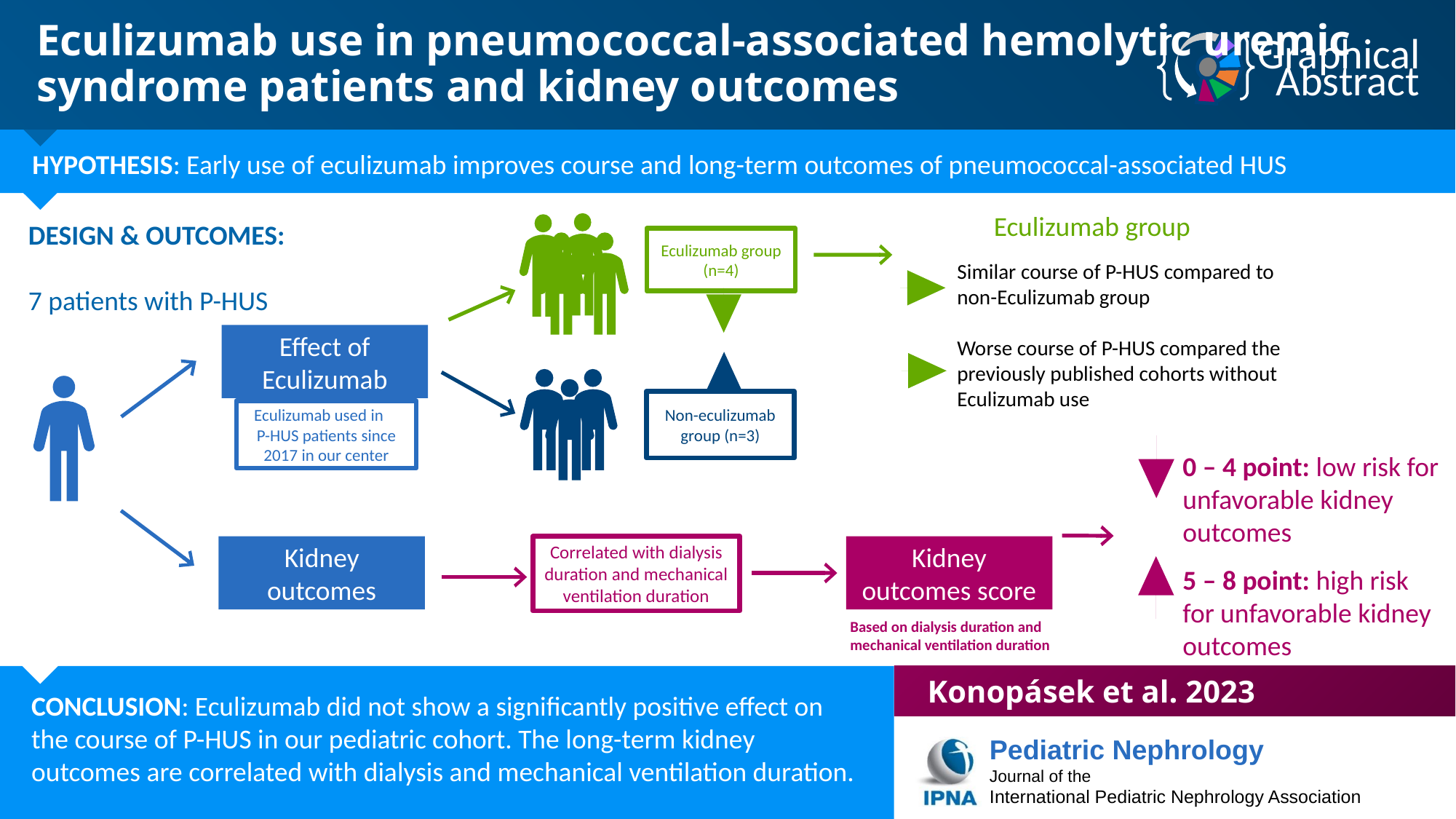

Eculizumab use in pneumococcal-associated hemolytic uremic
syndrome patients and kidney outcomes
HYPOTHESIS: Early use of eculizumab improves course and long-term outcomes of pneumococcal-associated HUS
Eculizumab group
DESIGN & OUTCOMES:
7 patients with P-HUS
Eculizumab group (n=4)
Similar course of P-HUS compared to non-Eculizumab group
Worse course of P-HUS compared the previously published cohorts without Eculizumab use
Effect of Eculizumab
Non-eculizumab group (n=3)
Eculizumab used in P-HUS patients since 2017 in our center
0 – 4 point: low risk for unfavorable kidney outcomes
Kidney outcomes
Kidney outcomes score
Correlated with dialysis duration and mechanical ventilation duration
5 – 8 point: high risk for unfavorable kidney outcomes
Based on dialysis duration and mechanical ventilation duration
Konopásek et al. 2023
CONCLUSION: Eculizumab did not show a significantly positive effect on the course of P-HUS in our pediatric cohort. The long-term kidney outcomes are correlated with dialysis and mechanical ventilation duration.
